# Contribution to Understanding of Synergy between Red Mud and Common Supplementary Cementitious Materials

**DOI:** 10.3390/ma15051968

**Published:** 2022-03-07

**Authors:** Ivana Vladić Kancir, Marijana Serdar

**Affiliations:** Department of Materials, Faculty of Civil Engineering, University of Zagreb, HR-10000 Zagreb, Croatia; ivana.vladic.kancir@grad.unizg.hr

**Keywords:** red mud, fly ash, slag, limestone, calcined clay

## Abstract

Recently, much attention has been paid to the reuse of bauxite residues from alumina production, also known as red mud, in the cement industry. Red mud bears the potential to improve concrete properties due to its favourable chemical composition and particle size. In this work, the synergy between locally available red mud and common supplementary cementitious materials such as fly ash, slag, calcined clay and limestone in cement mixes is investigated. All materials used were sourced from the immediate vicinity of the cement plant. The study of synergy involved the evaluation of the individual chemical reactivity of each material using the R3 test by isothermal calorimetry as well as their joint contribution to the heat of hydration and the composition of the reaction products of the paste and the compressive strength of the mortar. The results show how, by understanding the synergy between the materials, a higher level of cement substitutions can be achieved without compromising the mechanical properties of the mortar.

## 1. Introduction

The improvement of the standard of living and the progress of industrial activity led to an increased amount of waste produced by society. The quantities of industrial wastes that accumulate in landfills and pose a serious environmental problem can be reduced through recycling. At the same time, one of the main strategies employed by the cement industry to reduce its environmental impact is the use of supplementary cementitious materials (SCMs), which are mostly obtained as by-products and waste materials from various industries. Materials such as fly ash and granulated blast furnace slag have been used in the blended cements for many years. However, their availability is limited, especially the availability of fly ash in Europe, due to the coal phase-out strategy [1,2].

In recent decades, bauxite residue or “red mud” received some attention along with the studying of uncommon supplementary cementitious materials [3,4,5,6]. Red mud is a waste generated during Bayer’s process of alumina production. The Bayer process represents the primary method of producing alumina from bauxite where bauxite ore is dissolved in sodium hydroxide [7]. This highly alkaline product with pH 10–13.5 is pumped away for disposal and thus representing a great environmental threat [1,8].

Due to a very fine particle size, high alkalinity, and high iron content red mud application remains very limited [9]. Certain studies explored the possibility of using red mud in the construction industry [8,10,11]. In a study performed by Ribeiro et al. [12] it is shown that untreated red mud does exhibit pozzolanic properties according to Brazilian standard NBR 5751 and NBR 5752. However, the 28-day strength of mortars when the replacement level changed from 5 to 50%, decreased. Therefore, it was concluded that mortars with higher replacement of cement by red mud can be used for non-structural applications [12]. Still, it was hypothesised that using bauxite residue as an additive can improve the durability of concrete by increasing the resistance to chloride penetration and carbonation due to pore refinement [13]. Additionally, it was proven that bauxite residue can inhibit corrosion in reinforced concrete [14].

Although red mud, due to its lack of reactive silica, exhibits lower reaction than fly ash or slag, the idea of its use in construction materials is still present, also in synergy with other SCMs in cementitious or alkali-activated binders. [12,15,16,17]. High alkali content in red mud can promote hydration of Portland cement [12] and could help activate SCMs such as slag, fly ash or limestone. Moreover, it is reported that slag dissolution can be activated by increasing the pH value of the solution [18].

The literature review revealed that red mud bears potential as an SCM, but with very limited intrinsic reactivity. Therefore, the hypothesis of this study is that a synergistic effect between red mud and limestone, fly ash and silica fume would prove more promising due to the high alkalinity of red mud. It was unclear in the literature which materials achieve the highest synergistic effect when used together with red mud. In this study, the synergy of various supplementary cementitious materials (SCM) in cement pastes was analysed. In all systems, 20% of the cement was replaced with red mud. In addition to red mud, fly ash, slag, clay, and limestone were also employed in the mixes to observe the hydration of such composites. All used materials were obtained as waste and by-products from industrial processes in close vicinity of the cement production plant, such as aluminium production and thermal power plants in Bosnia and Herzegovina and stone quarry and clay excavation sites in Croatia [19].

## 2. Materials and Methods

### 2.1. Materials

**Figure 1:**
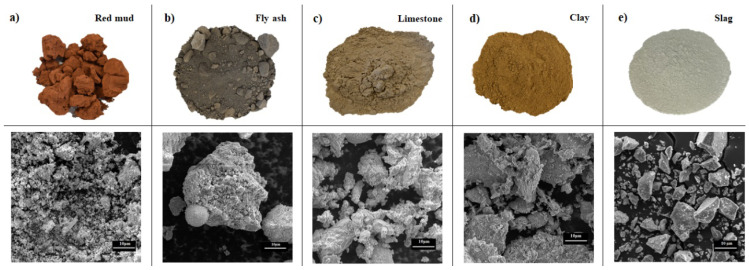
Visual appearance of as-collected raw materials (top image) and their particle appearance obtained with SEM (bottom image) used for morphological comparison: (**a**) red mud (RM), (**b**) fly ash (FA), (**c**) limestone (LS), (**d**) clay (C), (**e**) slag (SL).

To analyse the synergetic effect between red mud and other supplementary cementitious materials, following materials from the region were used: red mud (RM) from alumina production in Dobro Selo, Bosnia and Herzegovina, fly ash (FA) from a coal combustion thermal power plant in Tuzla, Elektroprivreda, Bosnia and Herzegovina, slag (SL) obtained as a final product from the Holcim, Koromačno, Croatia cement production plant, waste limestone powder (LS) from the quarry in Zvečaj, Arkada, Croatia and clay (C) from the brick production in Cerje Tužno, Croatia. Clay sample (C) before analysis was calcinated at 800 °C for 1 h. SCMs were used in combination with commercial cement, CEM 1 42.5 N, produced by Holcim Koromačno, Croatia. Figure 1 shows the visual appearance of raw materials and their microscopic images obtained by scanning electron microscope, SEM (Tescan Vega III Easyprobe microscope (Tescan, Brno-Kohoutovice, Czech Republic).

The differences in materials in terms of morphology and particle size can be clearly seen. SEM image of red mud shows very fine irregular particles. Morphology of all particles is mainly irregular, but in the SEM of fly ash, regular spherical shapes are visible. Both fly ash and slag particles form conglomerates. Image of clay displays even bigger irregular particles than rest of materials, also with a tendency to form conglomerates. The chemical compositions of used materials obtained using X-ray fluorescence (XRF) (Nex CG, Rigaku, TX, USA) are reported in Table 1.

**Figure 2:**
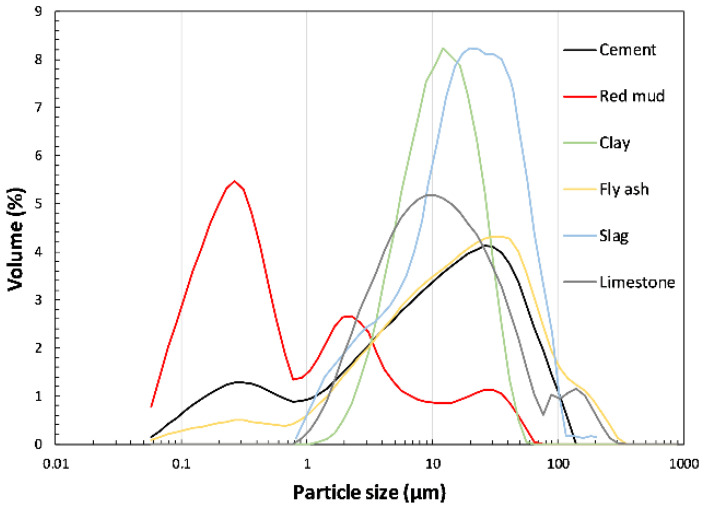
Particle size distribution of used materials obtained by laser diffraction.

The red mud sample contains a considerable amount of iron oxide and highest content of sodium oxide compared to other used materials which is connected to its origin. Fly ash and clay samples are rich in silicon dioxide and alumina. Both materials have a sum of pozzolanic oxides (SiO_2_, Al_2_O_3_ and Fe_2_O_3_) higher than 80% according to the European standard for fly ash for concrete EN 450:1 [20]. Considering C618—19ASTM [21] fly ash is classified as class F fly ash with a calcium oxide amount lower than 18% and SO_3_ amount less than 5%. Slag is rich in silicon dioxide and contains a considerable amount of calcium oxide. Most material samples have a sum of pozzolanic oxides higher than 70% concerning EN 450:1 [20] except limestone powder which is mostly composed of calcium carbonate. The particle size distribution in volume fraction obtained by laser diffraction (Mastersizer 2000 instrument (Malvern Panalytical, Malvern, UK)) is given in Figure 2.

Red mud sample bears a wide distribution of particles with highest numbers in the range of 0.1–1 μm. Compared to other materials, where most particles are between 1 and 100 μm, red mud particles are finer. Fly ash, limestone and ordinary Portland cement (OPC) sample also have a relatively wide distribution but highest number of particles is around 100 μm. Unlike fly ash and OPC, the clay and slag samples exhibit a different range of particle sizes with higher volume frequency. The median *d*_50_ diameter and the specific surface area are given in Table 2.

The differences between materials regarding particle size are most clearly seen. Except for the reference mixture, all cement pastes were prepared with a high degree of cement substitution. In each mixture, 20% of the OPC was replaced by red mud. Additional 15% and 20% of cement was replaced with fly ash, slag, and clay, to investigate the synergic effect of different SCMs. To compare the effect of limestone on cement mixes with mixes containing common SCMs, 5% limestone was added. Each mix was blended according to the standard EN 196-1 [22] with a water to binder ratio of 0.5. The mixing ratios of the cement mixtures are given in Table 3.

### 2.2. Paste and Mortar Mixes

All mixtures were prepared with cement replacement with 20% by red mud (RM) and then additionally with 15 or 20% by fly ash (FA), slag (SL) and clay (C). Additionally, in some mixtures 5% of limestone (LS) was added as a supplementary cementitious material. Therefore, cement replacement levels rose from 20 to 45%. Mixes were labelled with percentage of replacement level in front of acronym of material used in mix. For example, mix labelled 20RM20FA5LS contains 20% of red mud, 20% of fly ash and 5% of limestone and wt. (%) of cement in this mix is 60%. All mixtures were prepared with the same 0.5 w/b ratio.

### 2.3. Methods

Before preparing cementitious mixtures, red mud and clay samples were dried at 60 °C until constant mass, and ground in a ball mill for 2 min. Additionally, clay was calcined at 800 °C for 1 h. Other materials were used in mixtures as received, since they did not require further treatment. The individual chemical reactivity of each SCM material was determined in a simplified cementitious system by R3 test according to ASTM standard C1897—20 [23]. The test was carried out at 40 °C by isothermal calorimetry. In R3 test, firstly, SCM material is blended and homogenized with calcium carbonate and then mixed with potassium solution in order to achieve Portland cement environment. Before mixing, dry mixture of SCM, potassium solution and glass calorimetry vials are stored in 40 ± 2 °C environment until the temperature of dry mixture is stabilized. The reactivity was evaluated by measuring the total heat of hydration released after 3 days of testing.

The heat of hydration of pastes with different binder compositions was monitored for 3 days at 20 °C using an 8-channel TAM Air isothermal calorimeter (TA Instruments, New Castle, DE, USA). For each mixture, 10 g of fresh paste at a ratio of 0.5 w/b, mixed outside the calorimeter, poured into glass vials and then placed in the calorimeter. Thermogravimetric analysis (TGA) was performed using TGA 55 (TA instruments, New Castle, DE, USA) in the temperature range of 40 °C to 1000 °C with a constant heating rate of 20 °C and a nitrogen flow of 40 mL/min. TGA was performed on 50 ± 5 mg of finely ground powder from mortar samples after 2, 7 and 28 days to follow the formation of hydration products. The test was performed on the exact age and hydration was not stopped prior to testing. Compressive strength test of the mortars was performed on 40 mm × 40 mm × 160 mm prisms according to EN 196-1. After 24 h, the specimens were demoulded and cured in a humidity chamber (20 °C and 95% RH) until testing after 2, 7 and 28 days.

## 3. Results

### 3.1. Individual Reactivity of Used SCMs

**Figure 3:**
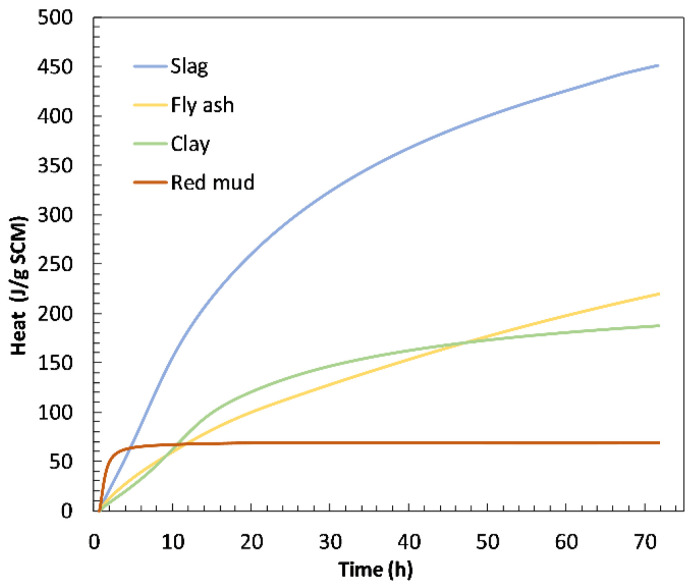
Reactivity of materials acquired by R3 test using isothermal calorimetry.

**Figure 4:**
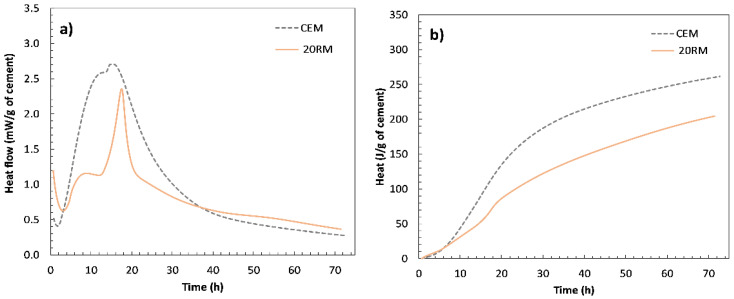
Heat flow (**a**) and cumulative heat curves (**b**) of reference paste (CEM) without substitution and of paste (20RM) with 20 wt.% red mud (RM) obtained by isothermal calorimetry.

Figure 3 displays the reactivity of the materials used in this study, which was determined by the R3 test method using isothermal calorimetry. Slag showed the highest heat release over 3 days of testing (total heat 451 J/g SCM) and was the most reactive material among those shown in Figure 4. The red mud sample showed the highest initial reactivity, after which the heat remained constant and showed the lowest heat release after 3 days (total heat 69.3 J/g SCM). Fly ash and clay exhibited similar behaviour after three days. In the first 45 h, clay sample showed higher reactivity than fly ash, but after 45 h, reactivity of fly ash was higher. Fly ash sample developed a higher heat of hydration (220 J/g SCM) than clay sample (190 J/g SCM) after three days.

### 3.2. Analysis of Red Mud-Cement System

Figure 4 shows the heat of hydration of the reference sample (OPC) paste without any substitution and the paste with 20 wt.% cement substituted by red mud (RM) measured by isothermal conduction calorimetry during 72 h. In Figure 4a,b, heat flow curve and cumulative heat curve are shown, respectively. The plots are normalised to Portland cement content in the mortar, i.e., to mass of cement, to allow clear differentiation of the contribution of the red mud to the heat evolution. In a typical isothermal calorimetry curve for the hydration of ordinary Portland cement, the first peak generally corresponds to the hydration of the silicate phases, while the second peak is generally associated with the reaction of the aluminate phases [24].

In the system with red mud, two peaks occurred after the induction phase. As can be seen in Figure 4a,b, the second peak was more pronounced than in the OPC system and occurred after the main peak associated with hydration of the alite [25]. A similar heat flow curve was observed for certain fly ash systems [26]. In the case of fly ash, the increase in the second peak was attributed to the reaction of tricalcium aluminate (C_3_A) and the subsequent conversion of ettringite to monosulfate, as fly ash provides a high number of nucleation sites for the hydration products of calcium aluminate. Since the particle size of the red mud is even finer than that of the fly ash (Figure 2), the increase in the second peak could be due to the high proportion of very fine particles providing nucleation sites for the precipitation of the aluminate phases. A similar distinguished second peak was also observed in the hydration studies of calcium aluminate cement [27]. In their study of a low calcium sulphate system, the second more accentuated peak coincided with an accelerated calcium aluminate (CA) reaction and gypsum depletion, followed by ettringite consumption and monosulfoaluminate formation [27]. The similarity of hydration results could be due to the influence of red mud, which contains a higher amount of alumina than cement. The alkali content in the red mud is higher than that in the OPC system, which could additionally advance the second peak of hydration. From the literature it was observed that the hydration of calcium aluminate can be affected by the presence of alkalis [28].

**Figure 5:**
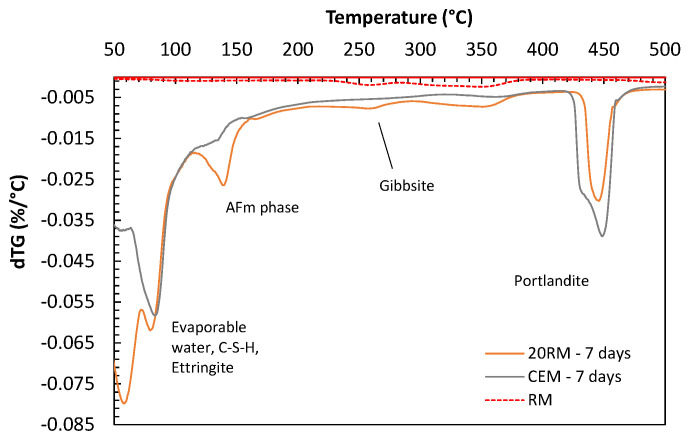
Derivative of mass loss (dTG) of cement mortar mixes, reference (OPC) and mix with 20 wt.% cement replacement with red mud after 2 and 7 days of curing.

Formation of aluminate phases was confirmed with TGA performed after 7 days of hydration, Figure 5, where a peak between 110–160 °C for system with red mud could be explained by the formation of aluminate AFm phases [1,29]. The AFm peak in the case of cement with red mud is significantly more pronounced compared to OPC system.

Furthermore, the first peak between 60 °C and 100 °C observed with TGA seems enlarged for mix with red mud compared to OPC. In this range, general decomposition of hydrated compounds of silicates, sulfates and aluminates could be expected and here this peak could be attributed to ettringite, CSH and CAH gel formed in the blend with cement and red mud. Higher alkali content can also improve alkali sulfate formation with sulfates present in clinker [30].

### 3.3. Analysis of Red Mud-Fly Ash-Limestone Synergy

**Figure 6:**
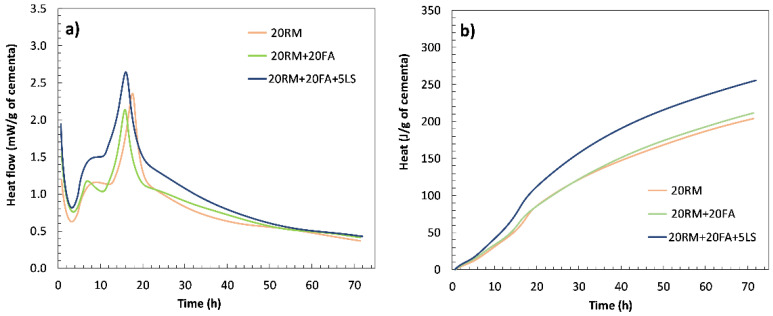
Heat flow (**a**) and cumulative heat curves (**b**) of the systems with red mud and fly ash (without and with the addition of limestone) obtained by isothermal calorimetry for 72 h.

First, the synergic effect of red mud and fly ash was analysed, as well as additional effect of limestone. Figure 6a,b shows the heat of hydration of pastes where 20 wt.% of cement was substituted with red mud and additional 15 and 20 wt.% was substituted with fly ash without and with limestone (5 wt.%).

When fly ash was added to a mixture with red mud, the shape of the calorimetry curve was similar to that of the paste with red mud only, the second peak being more pronounced. The addition of fly ash provides more alumina to the system. Although the shape of the calorimetry curve was similar, in the mixtures with red mud and fly ash, the first peak was modified and well pronounced after the induction time. When 5 wt.% limestone was added, both the first and second peaks increased, and the cumulative heat release also increased. The chemical reaction between fly ash, red mud and limestone occurs potentially due to the interaction between calcium carbonate from limestone and aluminate hydrates formed by OPC and red mud/fly ash, which was present in literature already observed with limestone and alumina rich cementitious materials [31]. Limestone accelerates and amplifies the aluminate reaction as indicated by the second peak in the isothermal calorimetry [31]. Similar to systems with fly ash and limestone, mono- and hemicarboaluminate hydrates could be formed here instead of calcium sulfoaluminate, leading to stabilisation of ettringite and an increase in the total volume of hydration products [32]. Previous studies have shown that this synergy between fly ash and limestone leads to a decrease in porosity and positively affects compressive strength [32].

**Figure 7:**
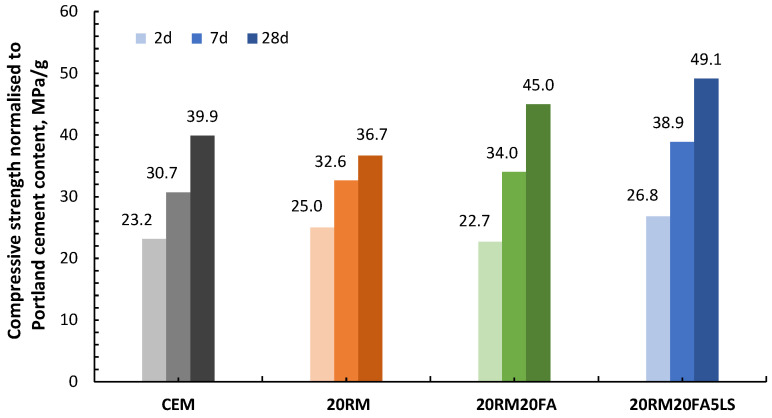
Compressive strength normalised to Portland cement content at 2, 7 and 28 days of mortars with 20 wt.% cement substitution by red mud, with additional 20 wt.% cement substitution by fly ash, without and with 5 wt.% cement substitution by limestone.

The calorimetry results show a similar trend as the results of compressive strength, which are shown in Figure 7. The compressive strength results are presented per mass of cement, i.e., they are normalised to Portland cement content in the mortar. The reason for such presentation of data is to enable clear distinction of the influence of each cement substitution. The logic behind this presentation is the same as with the normalisation of heat of hydration data. If SCM used is fully inert, the values of compressive strength normalised to Portland cement content would be the same for OPC and for the mix with substitution. On the other side, if SCM used is reactive and therefore contributes to the development of strength, the compressive strength normalised to Portland cement would be higher, since it is not only cement in the mix contributing to the strength. From the results of compressive strength normalised to Portland cement shown in Figure 7, it can be observed that the addition of red mud resulted in a slight increase in the compressive strength in the early stages of 2 and 7 days, compared to the OPC system. However, in later stages the strength per gram of cement decreased due to the addition of red mud. This trend in compressive strength with the addition of red mud is consistent with the reactivity of red mud observed in the R3 test (Figure 3). The red mud started to react very quickly, which can be observed from the increase in the heat of hydration (Figure 3) and the compressive strength result after 2 days (Figure 7). However, after this initial reactivity, the red mud behaves inert, which is evident from both the reactivity test, Figure 3, and the results of the compressive strength after 28 days, Figure 7. Once fly ash was added to the system, the strength at early stages remained similar but there was a significant contribution to the later strength at 28 days. Finally, when additional limestone was added, both early and late strength were increased, proving a positive synergy between red mud, fly ash and limestone.

### 3.4. Analysis of Red Mud-Slag-Limestone Synergy

**Figure 8:**
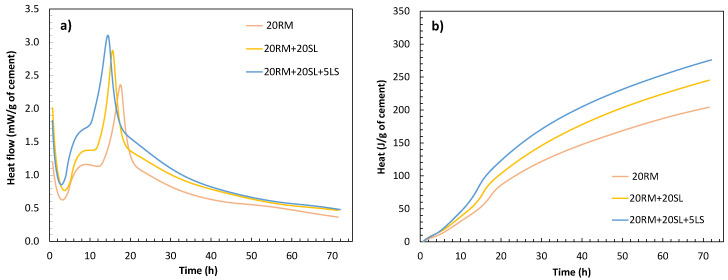
Heat flow (**a**) and cumulative heat curves (**b**) of the systems with red mud and slag (without and with the addition of limestone) obtained by isothermal calorimetry for 72 h.

Next, synergic effect of red mud and slag was analysed, as well as additional effect of limestone addition. Figure 8 shows the heat of hydration of pastes where 20 wt.% of cement was substituted with red mud and additional 15 and 20 wt.% was substituted with fly ash without and with limestone (5 wt.%).

When slag was added instead of fly ash, both the first and second peaks of hydration and the cumulative heat release increased. This indicates a better synergistic effect between slag and red mud than between fly ash and red mud. Red mud in the system increases the medium alkalinity and potentially accelerates slag dissolution [16,33]. In addition, slag alone has higher reactivity than other SCMs used as shown in Table 2. In contrast to the system with only red mud, the inclusion of slag enhanced the first hydration peak, probably because the slag accelerates the hydration of the C_3_S phase [16,34,35]. The aluminate reaction was also enhanced since the slag has an additional alkali content, and as mentioned earlier, alkalis promote the hydration of aluminates.

**Figure 9:**
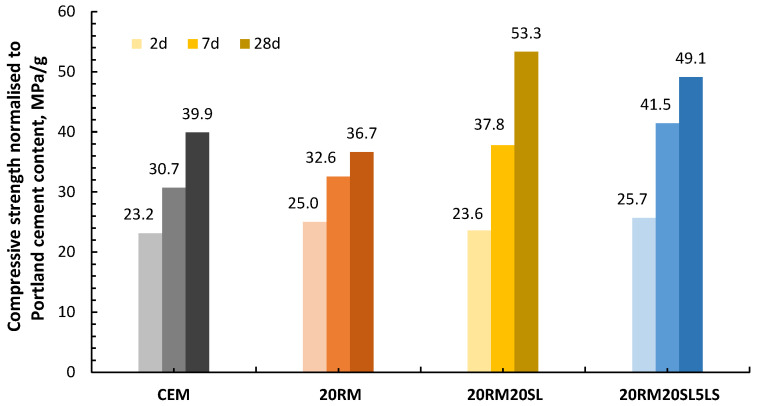
Compressive strength normalised to Portland cement content at 2, 7 and 28 days of mortars with 20 wt.% cement substitution by red mud, with additional 15 and 20 wt.% cement substitution by slag, without and with 5 wt.% cement substitution by limestone.

From the results of compressive strength per mass of cement, Figure 9, it can be seen that the addition of slag in the system helps to increase the compressive strength, especially at later stages. In addition, the presence of limestone increased the early strength, which is consistent with the increased heat of hydration. Such an influence of limestone on early strength increase was also reported in other studies [36]. However, the presence of limestone did not lead to an increase in strength after 28 days, as was the case in systems with fly ash. The absence of the effect of limestone on compressive strength after 28 days could be due to the fact that slag bears a lower alumina content than other materials [37].

### 3.5. Analysis of Red Mud-Calcined Clay-Limestone Synergy

**Figure 10:**
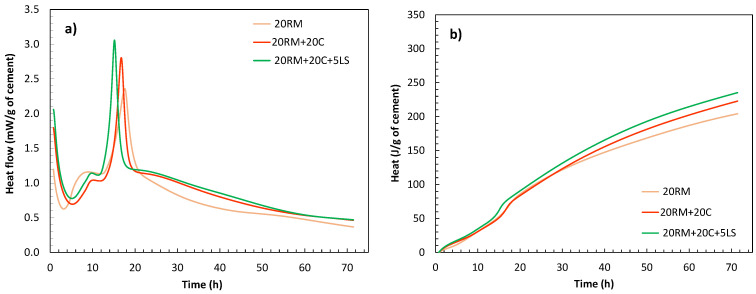
Heat flow (**a**) and cumulative heat curves (**b**) of the systems with red mud and calcined clay (without and with the addition of limestone) obtained by isothermal calorimetry for 72 h.

Finally, the synergistic effects of red mud and clay and the additional effect of limestone addition were also analysed. Figure 10 shows the heat of hydration of pastes where 20 wt.% of the cement was replaced by red mud and additional 15 and 20 wt.% by clay without and with limestone (5 wt.%).

**Figure 11:**
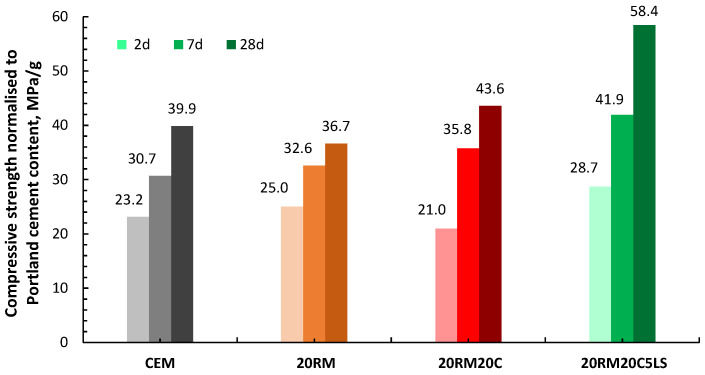
Compressive strength normalised to Portland cement content at 2, 7 and 28 days of mortars with 20 wt.% cement substitution by red mud, with additional 15 and 20 wt.% cement substitution by clay, without and with 5 wt.% cement substitution by limestone.

The addition of clay did not contribute to the development of the first hydration peak. It was discovered that the first hydration peak depends on the chemical reactivity of the materials (Table 2), with the most reactive slag showing the highest first peak and the clay with the lowest reactivity showing the lowest peak. The calcium aluminate reaction and thus the second peak seem to be enhanced with clay, probably due to the synergy with red mud, where the alkali content is even higher than in other systems. In the mixture with limestone, both the heat flux peak and the cumulative heat were enhanced. The compressive strength results of mortars containing red mud, clay and limestone are shown in Figure 11 and are presented per mass of cement.

From the compressive strength results per mass of cement, it can be seen that the addition of clay to red mud causes an even greater decrease in the early strength of the mortar. The synergy of clay and red mud appears to occur only at later stages (after 7 and 28 days), where their contribution to strength is positive. When 5% of limestone was added to the red mud and clay mixture, the compressive strength increased, which was especially evident after 28 days. A small amount of limestone increased the synergy between red mud and clay. Once limestone is added to the mix with high alumina red mud and clay and the mix is optimized, limestone improves the mechanical properties, due to its filling effect and the additional amount of calcium interacting with the alumina hydrates [38]. In systems with clay and limestone, heat of hydration and compressive strength are in good correlation.

### 3.6. Analysis of Reaction Products

**Figure 12:**
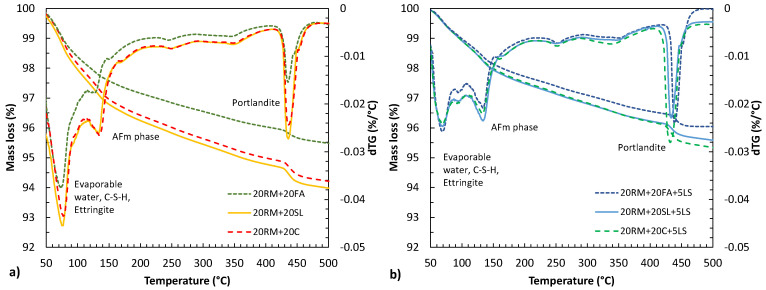
Mass loss and derivative of mass loss (dTG) for mortar mix with: (**a**) 40% cement replacement by 20% red mud and 20% fly ash or 20% slag or 20% calcined clay, (**b**) 45% cement replacement by 20% red mud, 5% of limestone and 20% fly ash or 20% slag or 20% calcined clay.

Difference in hydration products occurring in blended mixes were analysed using TGA. Figure 12 shows derivative of mass loss (dTG) of mortar mixes with: (a) 40% cement replacement by 20% red mud and 20% fly ash or 20% slag or 20% calcined clay, and (b) 45% cement replacement by 20% red mud, 5% limestone and 20% fly ash or 20% slag or 20% calcined clay.

The first main peak observed in all mixes ranged between 50 °C and 100 °C and could be attributed to the decomposition of C-S-H, CAH and ettringite [29,32,39]. This peak appeared to decrease with the addition of limestone to the mixture, especially with prolonged curing. The second main peak appeared in the range between 110 °C and 160 °C. In the reference sample, Figure 5, without clinker replacement, this peak was not as pronounced as in all other mixes. This could indicate the formation of AFm phases [40]. When comparing fly ash to clay and slag mixes on Figure 12a, it can be observed that the AFm peak was less pronounced, which is in accordance with the lower compressive strength of the mix with red mud and fly ash. With the addition of limestone, the curve changed, and most likely the limestone caused the formation of calcium carboaluminate instead of calcium sulfoaluminate, which could provide an explanation for the increased compressive strength [41]. The third peak between 400 °C and 500 °C is attributed to the dehydroxylation of portlandite. With higher cement replacement, the volume of hydration products decreases. This is especially the case with Portlandite peak, which decreased in the system with more fly ash.

A clear understanding of the synergy between different SCMs is only possible if the reaction products and their quantities are revealed. The TGA method used in this study contains certain limitations in clearly distinguishing the different hydration products. It is therefore necessary, as a continuation of this research, to perform a detailed analysis of the hydration phases using XRD to confirm the formation of the phases mentioned in the manuscript (such as carboaluminate).

## 4. Discussion

Red mud bears higher initial reactivity compared to other common SCMs used in this study, as evident from the reactivity test using isothermal calorimetry. However, after the initial reactivity, the heat stabilises, and the material behaves inertly. In addition to this initial reactivity, red mud contains a significant content of alkalis and a favourable particle size distribution. For this reason, the idea of the study was to combine red mud with common SCMs—such as fly ash, slag and clay to achieve a synergy between these materials that could overcome their individual shortcomings. Used fly ash, slag and clay are rich in alumina, while slag is additionally rich in calcium. In addition to these SCMs, limestone was added to activate additional synergy with materials rich in alumina.

Upon observing the heat evolution, it can be seen that the addition of alumina-containing materials to the cement enhances the second hydration peak, indicating the formation of alumina-based reaction products. The second peak in the heat of hydration curve was visible in systems with fly ash from previous studies [31], but not in such a pronounced form. With red mud in the system, this peak becomes more significant and dominant, even though red mud has a lower amount of alumina than fly ash, clay or slag. The reason for an interaction between red mud and fly ash/clay/slag could be explained by a higher alkali content in the red mud, since alkalis favour the reaction of alumina [28]. Moreover, in this study, the second peak was more pronounced in systems with clay and slag, both of which contain more alkalis than fly ash.

Limestone was added to enhance the synergy between red mud and common SCM and to target a reaction with reactive alumina within the SCM. In the presence of limestone, the heat flux and cumulative heat curves increased in all cases. The peaks in the hydration curve associated with the initial hydration products became more pronounced and the total heat increased compared to systems without limestone. All systems with added limestone had higher heat of hydration, and for the mixes with fly ash and clay, the increase in heat can be correlated with an increase in compressive strength. The increase in the first peak of the heat flow curve agreed with the individual reactivity of the SCMs. Namely, slag showed the highest chemical reactivity of all tested materials, and cement paste with slag exhibited the highest first peak. The reactivity of the clay sample measured the lowest, and the first peak also measured the lowest. With the addition of limestone, the early compressive strength per mass of cement was also higher in all mixes, indicating synergy between the materials. The addition of limestone had no positive effect on the 28-day strength of red mud and slag mortars, but it increased the 28-day strength of mortars with red mud and fly ash and clay. For the mixes with slag, it was seen that systems with red mud and slag already achieved high compressive strength per cement mass and that limestone did not further increase the strength. It could readily be seen that a combination of slag and red mud reached equilibrium, probably due to the lower proportion of alumina and the higher proportion of calcium in the slag. The addition of limestone was therefore unnecessary due to a lack of alumina with which to react.

**Figure 13:**
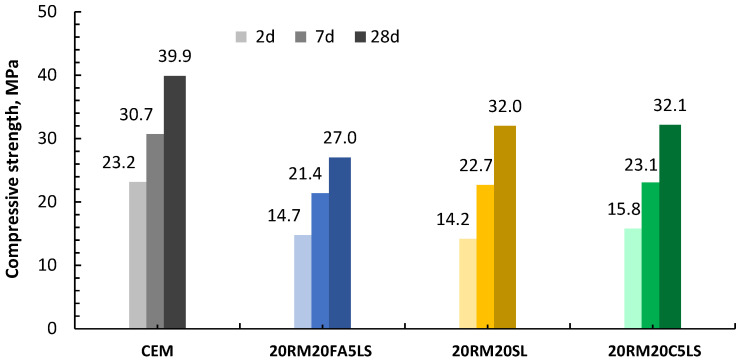
Compressive strength of mortars with optimised binders at 2, 7 and 28 days.

The synergy between the materials becomes clear when considering the results of absolute compressive strength, which are shown in Figure 13. While when limestone was added to red mud and slag, the compressive strength stagnated compared to the mixture without limestone, a significant increase in strength is observed when limestone is added to the system of clay and red mud. Finally, it can be observed that the mixture with red mud, clay and limestone containing only 45% cement reached 70% and 80% of the strength of the OPC system after 7 and 28 days, respectively.

In this study, there were two predominant parameters that controlled the reactivity and strength of the mixtures. The first parameter was the chemical composition, mainly the sum of pozzolanic oxides (SiO_2_, Al_2_O_3_ and Fe_2_O_3_), but more important was the sum of CaO and Al_2_O_3_ in the raw materials. It was observed that the mixtures with a better balance between CaO and Al_2_O_3_ achieved a higher compressive strength. Another parameter with great influence was the particle size distribution of binder obtained within the mixture. This parameter was found to mainly control the heat of hydration.

A clear understanding of the synergy between different SCMs is only possible if the reaction products and their quantities are revealed. It is therefore necessary to focus on the reaction phase assemblage in the next stage of this research, using both TGA and XRD.

## 5. Conclusions

In this study, the synergies between red mud and conventional SCMs were investigated. To identify any synergies between SCMs, the hydration process was monitored using isothermal calometry and correlated to the compressive strength of the mortar and the individual chemical reactivity of the SCMs, which was determined by the R3 test. Based on the results, the following conclusions can be drawn:

1. Red mud showed the highest initial heat release during the first two hours of the R3 test, as measured by isothermal calorimetry, when compared with other SCMs tested (slag, clay and fly ash). However, after the initial heat release, red mud behaved inertly throughout the test period.
2. A characteristic, distinct second peak in the calorimetry curve was observed in all systems containing red mud, which was attributed to the formation of aluminate phases. The formation of such phases was confirmed by TGA, with a distinct peak at temperatures around 150 °C.
3. Of all the combinations of red mud and SCMs tested, the highest synergy was obtained between red mud and clay with the addition of limestone. The highest synergy between these combinations of materials was attributed to the favourable particle size distribution of SCMs and the content of calcium, alumina and alkalis in these SCMs.
4. The mix containing the combination of red mud, clay and limestone, which consisted of only 55% of OPC, achieved 80% of the compressive strength of the pure OPC system after 28 days.
5. The addition of limestone improved the reactivity of alumina-rich SCMs, leading to a higher compressive strength of mixtures with fly ash and clay. In mixtures with slag, the addition of limestone did not improve compressive strength, possibly due to the lower percentage of alumina and the higher percentage of calcium already present in the slag.

A general conclusion based on the results presented is that by using the synergy of different SCMs, it is possible to increase the degree of cement substitution and thus overcome the individual deficiencies of each raw material without compromising mechanical properties of the mortar.

## Figures and Tables

**Table 1:** Chemical analysis of the used materials (wt.%).

Oxide	CEM I	Red Mud	Fly Ash	Slag	Clay	Limestone
SiO_2_	19.3	21.9	55.3	41.6	62.4	20.2
Al_2_O_3_	4.9	16.9	19.7	12.8	21.3	4.3
Fe_2_O_3_	2.9	37.9	9.0	6.0	7.3	1.4
CaO	64.0	10.0	8.3	33.5	2.2	71.6
MgO	1.8	0.6	2.9	6.0	1.8	1.7
SO_3_	2.8	0.2	1.4	1.6	0.1	0.1
Na_2_O	0.2	7.2	0.7	1.4	1.5	<0.01
K_2_O	0.8	0.2	1.7	0.6	2.5	0.1
P_2_O_5_	-	0.5	0.4	0.01	0.4	0.4

**Table 2:** Characteristic particle size, expressed as *d*_50_, and specific surface area of raw materials used.

Material	Particle Size *d*_50_ (μm)	Specific Surface Area (cm^2^/g)
I	9.9	3650
RM	0.4	8300
FA	15.2	5831
SL	20.8	4590
C	10.7	3146
LS	18	2524

**Table 3:** Mix proportions of cement composites.

Mix Label	Mass Ratio within the Binder (%)					
	CEM I	RM	FA	SL	C	LS
CEM	100	0	0	0	0	0
20RM	80	20	0	0	0	0
20RM15SL	65	20	0	15	0	0
20RM20FA	60	20	20	0	0	0
20RM20SL	60	20	0	20	0	0
20RM20C	60	20	0	0	20	0
20RM15SL5LS	60	20	0	15	0	5
20RM20FA5LS	55	20	20	0	0	5
20RM20SL5LS	55	20	0	20	0	5
20RM20C5LS	55	20	0	0	20	5

## Data Availability

Not applicable.
